# The Pass/Fail Effect: A Longitudinal Study of United States Medical Licensing Examination (USMLE) Step 1 Performance Over a Decade

**DOI:** 10.7759/cureus.41702

**Published:** 2023-07-11

**Authors:** Sweta Yadav, Anushka Dekhne, Samyuktha Harikrishnan, Babita Saini, Jooi Shukla, Tamara Tango, Yashasvi Patel, Mitkumar Patel, Raj Singh Chavda, Apurva Popat

**Affiliations:** 1 Internal Medicine, GMERS (Government Medical Education and Research Society) Medical College, Ahmedabad, IND; 2 Internal Medicine, American University of Antigua, St. John's, ATG; 3 Internal Medicine, Gulf Medical University, Ajman, ARE; 4 Internal Medicine, Uzhhorod National University, Uzhhorod, UKR; 5 Internal Medicine, Dr. M. K. Shah Medical College and Research Centre, Ahmedabad, IND; 6 Internal Medicine, Faculty of Medicine University of Indonesia, Jakarta, IDN; 7 Internal Medicine, Geetanjali Medical College and Hospital, Udaipur, IND; 8 Internal Medicine, MGM (Mahatma Gandhi Memorial) Medical College, Navi Mumbai, IND; 9 Internal Medicine, Smt. B. K. Shah Medical Institute & Research Centre, Vadodara, IND; 10 Internal Medicine, Marshfield Clinic, Marshfield, USA

**Keywords:** imgs, medical licensing examination, quality of medical education, medical school education, international medical school graduate, pass/fail usmle step 1

## Abstract

Objectives

This study aimed to analyze the impact of the United States Medical Licensing Examination (USMLE) Step 1 transition to a pass/fail scoring system in 2022 on the performance of first-time test takers in three distinct groups: Doctor of Osteopathy (DO) and Doctor of Medicine (MD) examinees from US/Canadian schools and examinees from non-US/Canadian schools. The analysis spans a decade-long period from 2012 to 2022, offering insights into the implications of this pivotal change in medical education.

Methods

We analyzed the performance of first-time USMLE Step 1 examinees from US/Canadian MD and DO programs and non-US/Canadian schools from 2012 to 2022, including the transition year to a pass/fail scoring system. Data were obtained from USMLE performance data reports and organized into annual contingency tables. Descriptive statistics and comparative analysis were used to identify trends and differences in performance across the groups. Data visualization techniques were employed to illustrate these findings, and the results were contextualized within the broader changes in medical education.

Results

In 2021, first-time takers from US/Canadian MD and DO Degree programs had pass rates of 96% and 94%, respectively, while non-US/Canadian schools had a pass rate of 82%. However, in 2022, these rates dropped to 93%, 89%, and 74%, respectively. The most significant relative decline was observed among non-US/Canadian Schools' first-time takers, with an 8% decrease. Repeaters consistently had lower pass rates across all groups.

Conclusion

The study reveals a notable decline in pass rates following the transition to pass/fail scoring, although this is based on just one year of data. This underscores the importance of students not rushing into the exam and dedicating sufficient time for preparation. The potential impact of this research could be transformative for medical education, but more years of data post-transition will be needed to confirm these initial findings. These findings serve as a reminder that the change in scoring does not diminish the rigor of the exam, prompting students to approach their studies with diligence and patience and potentially paving the way for systemic improvements in medical education and healthcare delivery worldwide.

## Introduction

The United States Medical Licensing Examination (USMLE) Step 1, a key milestone in medical education, transitioned to a pass/fail scoring system in 2022, sparking significant debate [1,2,3]. The primary objective of this transition was to reduce the overemphasis on Step 1 scores, which had become a high-stakes numerical value often overshadowing other essential aspects of a medical student's application for residency. The new system aimed to promote a more holistic review of residency applicants, considering a broader range of academic and personal attributes [1].

However, the implications of this transition are not fully understood, and it has raised several concerns. One of the main worries is the potential disadvantage for students from less well-known medical schools who may have previously relied on a high Step 1 score to stand out in the competitive residency selection process. Similarly, students who might have excelled with a high Step 1 score may be disadvantaged in the new system [2].

This study analyzed the impact of this transition on the performance of first-time test takers from US/Canadian and non-US/Canadian schools. The analysis spans a decade (2012-2022), providing insights into this significant change in medical education [4,5].

Our research is timely and relevant given the recent USMLE Step 1 scoring system changes. Understanding the potential impacts of these changes on student performance is crucial for the medical education community. The findings of this study will not only influence the quality of medical education but also have far-reaching implications for healthcare delivery worldwide. This is because the selection of residency candidates affects the quality of future physicians, which in turn impacts patient care [3,6].

The transition to a pass/fail system for the USMLE Step 1 is a complex issue with potential benefits and drawbacks. By examining the impact of this change on student performance, this study will provide valuable insights that can guide future policy decisions in medical education.

## Materials and methods

Data collection

The data for this study were obtained from the USMLE performance data reports spanning from 2012 to 2022. These reports provide comprehensive information on the performance of first-time USMLE Step 1 examinees from US/Canadian Doctor of Medicine (MD) and Doctor of Osteopathy (DO) programs and non-US/Canadian schools. The data include the number of examinees tested each year and the corresponding pass rates. The year 2022 was of particular interest as it marked the transition to a pass/fail scoring system for the USMLE Step 1.

Data analysis

The data were organized into annual contingency tables to facilitate analysis. Descriptive statistics were computed to provide a summary of the data and to identify trends over the 10-year period. Comparative analysis was performed to identify differences in performance across the three groups of examinees: US/Canadian MD, US/Canadian DO, and non-US/Canadian schools (Table 1).

The analysis focused on the pass rates of first-time test takers, as this group represents the majority of examinees each year. The pass rates for repeaters were also analyzed, although they represent a smaller proportion of the examinee population.

**Table 1:** Comparison of USMLE Step 1 performance: US/Canadian vs. non-US/Canadian candidates (2012-2022) MD: Doctor of Medicine; DO: Doctor of Osteopathy; USMLE: United States Medical Licensing Examination

	2012	2013	2014	2015	2016	2017	2018	2019	2020	2021	2022
Examinees from US/Canadian schools											
MD degree examinees	19,856	20,023	20,394	21,111	21,122	21,382	21,611	22,146	20,343	23,078	24,317
MD 1st-time takers	18,723	19,108	19,582	20,213	20,122	20,353	20,670	21,308	19,772	22,280	22,828
MD 1st-time takers pass rate %	96%	97%	96%	96%	96%	96%	96%	97%	98%	96%	93%
MD repeaters	1,133	915	812	898	1,000	1,029	941	838	571	798	1,489
Total MD pass rate %	94%	95%	95%	94%	94%	94%	95%	96%	97%	95%	91%
DO degree examinees	2,564	2,726	2,846	3,222	3,454	3,835	4,136	4,837	5,274	5,365	4,722
DO 1st-time takers	2,496	2,680	2,810	3,185	3,398	3,786	4,092	4,794	5,235	5,309	4,659
DO 1st-time takers pass rate %	92%	94%	93%	93%	94%	95%	96%	96%	96%	94%	89%
DO repeaters	68	46	36	37	56	49	44	43	39	56	63
Total DO pass rate %	91%	94%	93%	93%	93%	95%	96%	96%	95%	94%	89%
Examinees from non-US/Canadian schools											
Non-US total examinees	18,462	18,421	18,038	17,749	17,606	17,203	16,443	16,065	13,117	19,210	24,956
Non-US 1st-time takers	14,201	14,649	15,149	15,030	15,031	14,900	14,332	14,046	11,742	16,952	22,030
Non-US 1st-time takers pass rate %											
Non-US repeaters	4,261	3,772	2,889	2,719	2,575	2,303	2,111	2,019	1,375	2,258	2,926
Total non-US pass rate %	68%	72%	72%	72%	72%	73%	75%	78%	83%	77%	71%

Data visualization

Data visualization techniques were employed to illustrate the findings. Graphs were created to depict the trends in pass rates over time for each group of examinees. These visual representations facilitated a clearer understanding of the data and the impact of the transition to a pass/fail scoring system.

Contextualization

The results were contextualized within the broader changes in medical education, particularly the transition to a pass/fail scoring system for the USMLE Step 1. The implications of the findings were discussed in relation to the ongoing debate about the impact of this transition on medical education and residency selection.

Ethical considerations

As this study involved the analysis of publicly available, de-identified data, ethical approval was not required. All analyses were conducted in accordance with relevant guidelines and regulations.

## Results

Our analysis of the USMLE Step 1 performance data from 2012 to 2022 revealed significant trends among first-time test takers from non-US/Canadian schools, US/Canadian MD degree programs, and US/Canadian DO degree programs.

Non-US/Canadian schools

**Figure 1:**
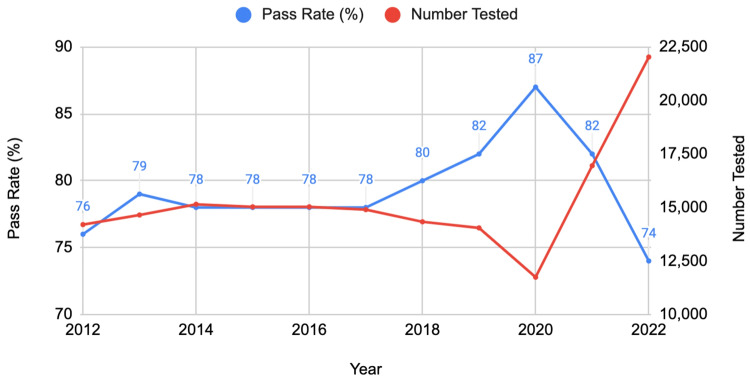
USMLE pass rates of non-US/Canadian schools' first-time test takers (2012-2022) USMLE: United States Medical Licensing Examination

For first-time test takers from non-US/Canadian schools, the pass rate fluctuated over the decade, peaking at 87% in 2020 and dropping to 74% in 2022, coinciding with the transition to a pass/fail scoring system. Concurrently, the number of test takers generally increased, indicating growing interest despite scoring changes. Interestingly, there was no direct correlation between the number of test takers and the pass rate (Figure 1).

US/Canadian schools (MD degree)

**Figure 2:**
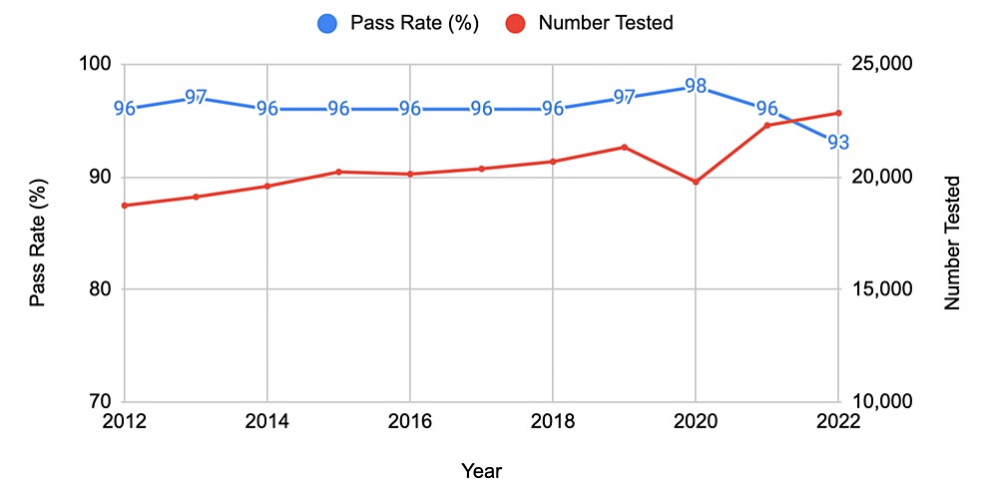
USMLE pass rates of US/Canadian schools' MD degree first-time test takers (2012-2022) USMLE: United States Medical Licensing Examination

For first-time test takers from US/Canadian MD degree programs, the pass rate remained relatively high over the decade, with a slight peak in 2020 at 98%. However, there was a noticeable drop to 93% in 2022. The number of test takers has generally increased over the years, with a significant rise from 19,772 in 2020 to 22,280 in 2021 and further to 22,828 in 2022 (Figure 2).

US/Canadian schools (DO degree)

**Figure 3:**
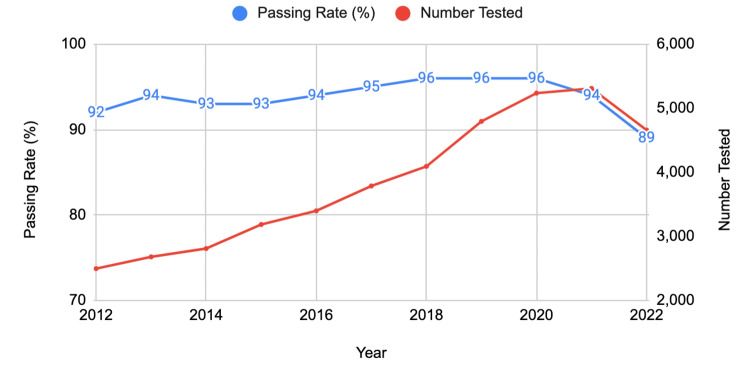
USMLE pass rates and number of examinees for US/Canadian DO degree first-time test takers (2012-2022) USMLE: United States Medical Licensing Examination

For first-time DO test takers from US/Canadian schools, the number of examinees showed a steady increase over the years, reaching a peak of 5,309 in 2021. This growth is indicative of the expanding role and recognition of osteopathic medicine within the healthcare system. However, this increase in the number of test takers was accompanied by a slight decrease in the passing rate, from 92% in 2012 to 89% in 2022 (Figure 3).

Comparative analysis

When comparing the three groups, the US/Canadian schools (MD degree) consistently demonstrated higher pass rates over the decade. However, all groups experienced a decline in pass rates in 2022, coinciding with the transition to a pass/fail scoring system for the USMLE Step 1. The most significant relative decline was observed among non-US/Canadian schools' first-time takers, with an 8% decrease from 2021 to 2022.

## Discussion

The medical education community has been significantly engaged in discussions following the transition of the USMLE Step 1 to a pass/fail scoring system in 2022 [6,7]. Our analysis of a decade-long performance data, spanning from 2012 to 2022, provides intriguing insights into this debate. We observed a notable decline in pass rates among all groups of first-time test takers, with non-US/Canadian schools experiencing the most significant relative drop. This finding underlines the crucial importance of adequate preparation and thoughtful timing when undertaking the exam.

This decline, however, could be attributed to several other contributing factors. Coinciding with the transition to a pass/fail scoring system, the exam's passing score was raised by two points to 196, potentially leading to the observed decline. An influx of students sitting the exam after the transition could have also played a role in the adjusted pass rates. Further reinforcing this point, a self-reported post-exam survey revealed a reduction in daily studying time and weeks of advance preparation in 2022 compared to previous years [8].

The initial intent behind the transition to a pass/fail scoring was to diminish the overemphasis on Step 1 scores, thereby promoting a more holistic evaluation of residency applicants [6]. The previous scoring system created what Chen et al. (2019) termed the "Step 1 climate" a high-stress environment with potential adverse effects on student well-being and learning [6]. Therefore, the observed decline in pass rates might reflect a shift away from this high-pressure climate [9].

Despite the change in scoring, the USMLE Step 1 remains a rigorous examination demanding significant preparation, as highlighted by Prober et al. (2016) [9]. Our findings serve as a stark reminder that despite a scoring transition, the stringency of the exam remains intact.

These observations offer valuable insights for future deliberations about the pass/fail transition and strategies for student preparation for the USMLE Step 1 exam. For instance, medical schools could provide students with clearer expectations for the exam under the new scoring system and offer more comprehensive support structures. As students navigate this transition, it is imperative to remind them that although the scoring system has changed, the need for diligence and sustained preparation remains undiminished.

Limitation

The study provides an initial analysis of the impact of the transition to a pass/fail scoring system based on just one year of data (2022). Future studies will be needed to confirm these initial findings and to explore the longer-term impacts of this transition.

## Conclusions

The USMLE Step 1's transition to a pass/fail system led to a decrease in pass rates, particularly among non-US/Canadian examinees, based on a year's data. This necessitates improved support for international students and a continued focus on diligent preparation. Although the change aims to promote a holistic review of residency applicants, the rigor of the exam persists. This underscores the need for enhancing student preparation, support systems, and equitable evaluation of applicants, influencing the future of medical education and healthcare worldwide.
